# Does Glucose-6-Phosphate Dehydrogenase Deficiency Correlate with Increased Sensitivity to Cisplatin? A Case Report and a Narrative Literature Review

**DOI:** 10.3390/ijms27093798

**Published:** 2026-04-24

**Authors:** Nerina Denaro, Valeria Smiroldo, Claudia Bareggi, Cinzia Solinas, Michele Ghidini, Massimo Castellani, Marco Carlo Merlano, Serafina Martella, Riccardo Giossi, Alessia Casbarra, Ornella Garrone

**Affiliations:** 1 Medical Oncology Unit, Fondazione IRCCS Ca’ Granda Ospedale Maggiore Policlinico Milano, 20122 Milan, Italy; valeria.smiroldo@policlinico.mi.it (V.S.); claudia.bareggi@policlinico.mi.it (C.B.); michele.ghidini@asst-settelaghi.it (M.G.); ornella.garrone@policlinico.mi.it (O.G.); 2 Medical Oncology Unit, University Hospital and University of Cagliari, 09042 Cagliari, Italy; czsolinas@gmail.com; 3 Medicina Nucleare Fondazione IRCCS Ca’ Granda Ospedale Maggiore Policlinico Milano, 20122 Milan, Italy; massimo.castellani@policlinico.mi.it; 4 Candiolo Cancer Institute, FPO-IRCCS Candiolo, 10060 Torino, Italy; mcmerlano@gmail.com; 5 Department of Biomedical and Biotechnological Sciences, University of Catania, 95123 Catania, Italy; seramartella@gmail.com; 6 SC Analisi Chimico Cliniche ASST Grande Ospedale Metropolitano Niguarda Milano, 20122 Milan, Italy; riccardo.giossi@policlinico.mi.it; 7 SC Radioterapia ASST Grande Ospedale Metropolitano Niguarda Milano, 20122 Milan, Italy; alessia.casbarra@ospedaleniguarda.it

**Keywords:** glucose-6-phosphate dehydrogenase (G6PD) deficiency, nasopharynx cancer, chemoradiotherapy, toxicity

## Abstract

Glucose-6-phosphate dehydrogenase (G6PD) deficiency impairs NADPH generation through the pentose phosphate pathway, resulting in reduced glutathione regeneration and increased vulnerability to oxidative stress. While its clinical significance is well described in hemolytic disorders, its impact on tumor biology and chemosensitivity remains poorly characterized. Cisplatin, a backbone agent in the management of nasopharyngeal carcinoma (NPC), exerts its cytotoxicity through the formation of DNA adducts and the robust induction of reactive oxygen species (ROS) activity. We report a patient with non-keratinizing NPC and a G6PD variant, a (class III) deficiency, who demonstrated a rapid and pronounced objective response to cisplatin-based induction and concurrent chemoradiotherapy. Unfortunately, the patient also exhibited signs of rapid and persistent hematologic (platelets and white cells) toxicity. Notably, no hemolytic events occurred. A narrative review of the available literature indicates that G6PD-deficient cells exhibit a reduced antioxidant reserve, increased cisplatin-induced DNA damage, and impaired activation of ROS-detoxifying pathways. A few clinical observations similarly report enhanced tumor responsiveness in G6PD-deficient individuals, although the evidence is sparse and heterogeneous. Preclinical data support the notion that diminished NADPH availability amplifies cisplatin-triggered oxidative injury, thereby increasing tumor susceptibility. This case adds to emerging evidence that G6PD deficiency may potentiate cisplatin efficacy in NPC by exploiting intrinsic redox vulnerabilities. While preliminary, these findings suggest the potential utility of metabolic phenotyping in treatment stratification. Prospective studies are needed to define the predictive value, safety, and therapeutic implications of G6PD status in cisplatin-based regimens.

## 1. Introduction

Beutler first described glucose-6-phosphate dehydrogenase (G6PD) deficiency in 1959, noting the particular sensitivity of some individuals to the hemolytic action of certain drugs [1].

G6PD deficiency is the most common human enzyme defect, affecting more than 400 million people worldwide [2].

The pentose phosphate pathway (PPP) is the sole source of nicotinamide adenine dinucleotide phosphate (NADPH) in red blood cells, which lack mitochondria; therefore, defense against oxidative stress in red blood cells depends on G6PD. So, reduced NADPH production leads to hemolysis (i.e., impaired regeneration of reduced glutathione) [3].

**Figure 1:**
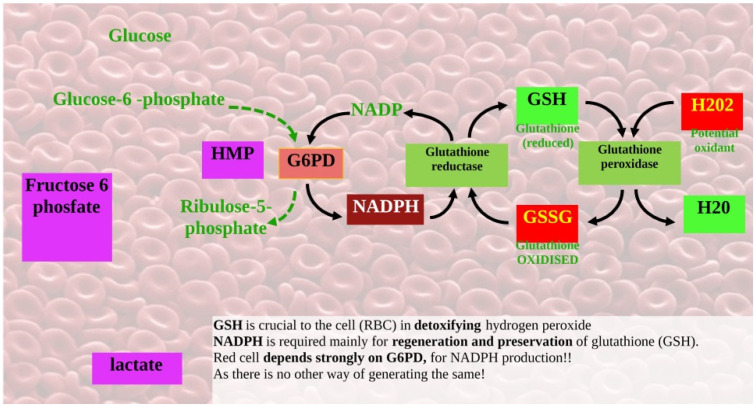
Glucose-6-phosphate dehydrogenase deficiency in red blood cells. G6PD deficiency can lead to the accumulation of lactate because the lack of the G6PD enzyme impairs the hexose monophosphate (HMP) shunt. The HMP pathway produces NADPH, a coenzyme essential for protecting red blood cells from oxidative damage. Elevated lactate levels are typical of a hemolytic crisis. When G6PD is deficient, the HMP shunt cannot generate enough NADPH to counteract oxidative stress caused by triggers such as certain medications, infections, or fava beans. Many chemotherapeutic agents (such as doxorubicin) increase oxidative stress as part of their mechanism of action.

In PPP, G6PD catalyzes the conversion of glucose-6-phosphate (G6P) into 6-phosphogluconolactone (6PGL), which then transforms into fructose-6-phosphate (F6P) and glyceraldehyde-3-phosphate (GADP), with the production of NADPH (Figure 1).

G6PD deficiency is an X-linked inherited genetic disorder caused by mutations in the G6PD gene, resulting in protein variants with varying levels of enzymatic activity, a state associated with a wide range of biochemical and clinical phenotypes. Indeed, there are more than 190 G6PD mutations described in the literature; most are point mutations that affect a single nucleotide. None of the mutation patterns detected in humans can result in complete inactivation of G6PD, since this is incompatible with embryo development.

The most common clinical manifestations of G6PD deficiency are neonatal jaundice and acute hemolytic anemia, usually triggered by an exogenous agent. Some G6PD variants cause chronic hemolysis, leading to congenital non-spherocytic hemolytic anemia. Impaired leukocyte function has also been documented, but it is rare [4].

The most effective way of managing G6PD deficiency is to prevent hemolysis by avoiding oxidative stress. G6PD deficiency confers resistance against malaria, as demonstrated by the similarity between geographic areas where G6PD is deficient and the Plasmodium falciparum malarial parasite is endemic (e.g., via oxidative stress disadvantages for Plasmodium-infected erythrocytes) [3,5].

Some G6PD variants are not associated with significantly reduced enzyme activity in erythrocytes; for example, G6PD A+ is not clinically relevant [6].

Hofmann et al. demonstrated that G6PD deficiency affects platelet aggregation; measurements obtained from platelet-rich plasma from deficient patients revealed an enhanced dose response to ADP, whose levels were one order of magnitude higher than in controls [7].

There is controversy about whether G6PD deficiency, without evidence of hemolysis, is associated with a lower platelet count compared with G6PD-normal. A recent retrospective study showed that the platelet count was higher in the O blood group–B blood incompatibility group compared to the O-A incompatibility group, but only when the G6PD level was >8.8 U/g Hb. A correlation between umbilical cord venous blood platelet count and G6PD levels was found only among O-B-incompatible female neonates [8].

There is an increasing amount of evidence indicating that G6PD is dysregulated in various diseases, such as cancers, blood hypertension, seizure disorders, and mental disease; G6PD dysfunction influences DNA synthesis, DNA repair, cell cycle regulation, and redox homeostasis, providing advantageous conditions for cancer cell growth, epithelial–mesenchymal transition (EMT), invasion, metastasis, and chemoresistance.

Here, we present a case report of a man with metastatic nasopharyngeal cancer who had a terrific response and grade 3 toxicities after only 1 cycle of chemotherapy.

The follow-up duration was 8 months because of geographic difficulties, as the patient resided in a different region.

Additionally, we present a non-systematic overview of G6PD deficiency in patients treated for cancer. The databases researched were Pubmed, Scopus, and Embase. The keywords used were “G6PD deficiency, cancer, nasopharyngeal cancer, chemotherapy, and radiotherapy”.

We selected 54 papers after excluding papers that were written in languages other than English and did not discuss cancer-related treatment. Ultimately, 28 papers were included.

## 2. Detailed Case Description

A 58-year-old man with a diagnosis of metastatic undifferentiated carcinoma of the nasopharynx that had extended to the vertebral column and cervical lymph nodes was referred to our oncology outpatient unit in June 2025.

His clinical history did not include any comorbidities, prior treatments, or other baseline clinical conditions that may influence hematologic tolerance.

He reported a G6PD class III deficiency (older classification) (variant A- according to the current WHO classification), without any signs or symptoms during his life. He had been tested after his brother developed neonatal jaundice.

The G6PD status was self-reported; later, we decided to correlate this condition with his response to chemotherapy.

In May 2025, an Ear, Nose, and Throat (ENT) examination revealed suspected pathological lymph nodes and a nasopharyngeal mass. A neck ultrasound demonstrated an increase in the number and size of multiple lymphadenopathies in the bilateral laterocervical regions. These lymphadenopathies were more pronounced on the left side. The lymph nodes exhibited a hypoechoic echotexture and altered vascularity. The largest node, located at level II on the left, had a short axis of approximately 1.6 cm. No abnormalities were observed in the thyroid glands. These findings were confirmed via neck and maxillary magnetic resonance.

Therefore, a fine-needle biopsy of the left laterocervical lymph node was performed. Histological examination showed fibroconnective tissue partially attributable to lymph node parenchyma infiltrated by a poorly differentiated neoplasm growing in nests and trabeculae. Immunohistochemical analysis revealed the following profile: pan-cytokeratin+, CAM5.2+ cytokeratin, cytokeratin 7−, cytokeratin 20−, GATA3−/+, PAX8–, NKX3.1−, CD30−, MUM1−, and EBV/EBER– (assessed via in situ hybridization). Immunostaining with anti-p40 antibody yielded an uncertain interpretation, as positivity in accessory cells could not be entirely excluded.

A nasopharyngeal biopsy was subsequently performed in June 2025. Histological examination confirmed a poorly differentiated non-keratinizing squamous cell carcinoma, which was EBV-positive (WHO 2022 classification), with immunohistochemical staining showing CK AE1/AE3+, p40+, and EBV/EBER+. At baseline, the Epstein–Barr virus (EBV) DNA level was 5470 copies (determined via a blood test on 11 June). At our institution, EBV DNA values range from <36 copies to >6000 copies.

In June 2025, a PET/CT scan showed an area of intense radiopharmaceutical uptake in the nasopharyngeal vault (SUV max 12), as well as other focal accumulations (SUV max: 13) in bilateral laterocervical lymph nodes (levels IIa, IIb, III, and IVa), with more extensive involvement caudocranially on the left side (levels IVb and Vb and more generally supraclavicular). The maximum sizes of the nodes were 39 × 23 mm on the right and 45 × 31 on the left. Skeletal uptake was observed in the vertebral segment of the left 3rd rib, anterior segment of the 4th rib, lateral segment of the ipsilateral 8th rib, and sternum. Additional focal accumulations were noted in the posterior segment of the right 10th rib, on the right apophyseal mass of the atlas, on the left pedicle of T5 and the right pedicle of S1, in the body of T11, on the transverse process of L1, in the bodies of L3 and L4, on the right iliac wing, and (smaller) on the left iliac wing. Of more uncertain but not necessarily less significance was the lymph node accumulation visible at the origin of the left external iliac vessels. The overall PET/CT findings were considered consistent with the presence of a primary lesion of the nasopharynx with associated widespread lymph node and skeletal metastases.

The patient started first-line chemotherapy on 30 June (following a blood test on 11 June). He received Cisplatin (75 mg/mq) and Gemcitabine (1000 mg/mq) on day 1 and 8. Thia cycle was prescribed every 21 days.

G6PD variant A was reported, although he was not retested, as his history did not say anything about hemolysis or any other G6PD signs or symptoms. Moreover, this information was added retrospectively due to severe hematologic toxicity. We asked for permission to perform further investigation, but the patient did not accept.

On day 8 of the first cycle, the EBV DNA level was 4557 copies (day 8 of therapy was postponed because of hematologic toxicities, as found in a blood test on 7 July).

After the first cycle of chemotherapy with cisplatin and gemcitabine, the EBV DNA level decreased to 351 copies. Unfortunately, the patient developed grade 3 hematologic toxicity (PLT 30,000 10^6^/L), which rapidly progressed to grade 4 (per the Common Toxicities Criteria for Adverse events v5.0 (CTCAE 5.0)).

Due to severe hematologic toxicity, particularly thrombocytopenia, the subsequent two cycles were postponed and later administered using weekly carboplatin instead of cisplatin and a reduced gemcitabine dose.

He underwent a second cycle with Carboplatin AUC 2 and Gemcitabine (750 mg/mq) on the 28th of July (28 days after the first cycle).

Following the second cycle, the EBV DNA level further decreased to <36 copies; however, hematologic recovery was not achieved.

Also, after the reduced doses of the second cycle were administered, the patient exhibited grade 3 hematologic toxicity according to the Common Toxicities Criteria for Adverse Events (CTCAE 5.0).

**Figure 2:**
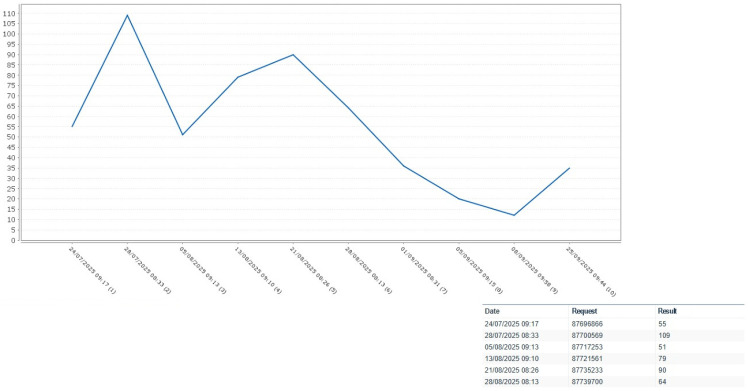
Platelets.

As the hematologic toxicities mainly affected the platelets, no growth factors were prescribed. In Figure 2 we reported haematologic toxicity.

This clinical case was discussed by the multidisciplinary tumor board team. We evaluated the patient’s clinical history and the clinical relevance of the G6PD variant. The patients did not have any signs or symptoms related to the disease. Moreover, we asked for permission to perform further investigation, but the patient refused.

With the aim of avoiding a definitive treatment interruption, the multidisciplinary team proposed sensitizing chemotherapy combined with radiotherapy. However, this strategy was not employed due to persistent hematologic toxicity.

In August 2025, a third cycle involving carboplatin AUC2 administered concurrently with radiotherapy was employed due to the patient’s platelet levels.

**Figure 3:**
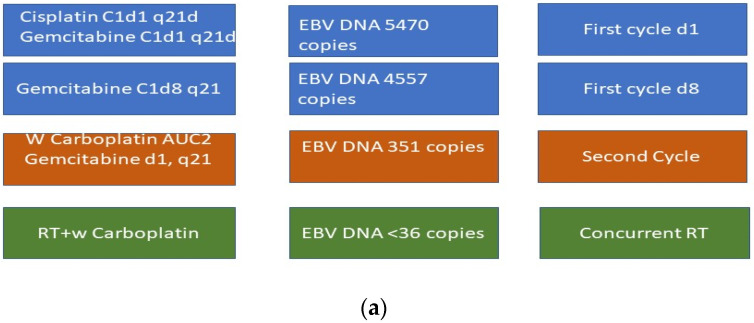

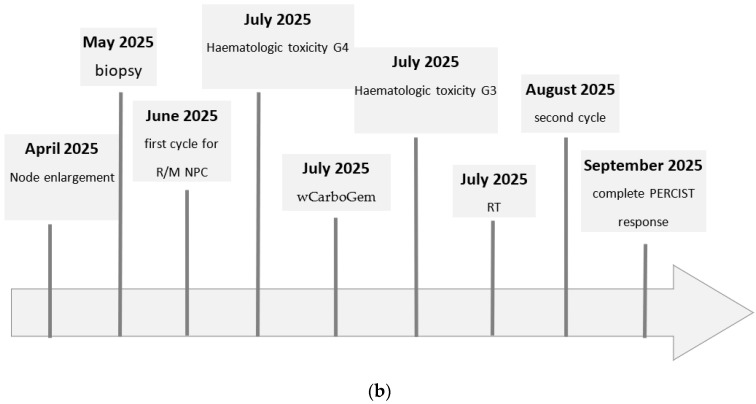
(**a**) Clinical flowchart. (**b**) Timeline.

During the 5 weeks of radiotherapy, EBV DNA became undetectable; however, the treatment was interrupted before completion (planned for 7 weeks) due to grade 3 dysphagia, mucositis, and radiodermatitis according to CTCAE5.0 (Figure 3a,b).

In order to exclude the presence of malignant cells in the bone marrow, a bone marrow biopsy was proposed, but the patient declined the procedure. A bone marrow biopsy is obtained using bone marrow aspirate, and it is a core biopsy for microscopic analysis, cytogenetics, and immunophenotyping for the diagnostic evaluation of benign and malignant hematological disorders, systemic diseases, infections, and non-hematological malignancies. We were looking for confirmation that there were no malignant cells in the bone marrow.

In Table 1, we report hematologic values observed during treatment.

Due to the absence of malignant cells in the bone marrow, the multidisciplinary team investigated other causes of marrow failure.

Throughout the course of treatment, no medications known to precipitate hemolysis in patients with G6PD deficiency were administered.

Fava beans and the drugs reported in Table 2 should be avoided by patients with G6PD deficiency.

A blood test was carried out to evaluate antibodies directed against platelet membrane glycoprotein (GP) complexes, GPIIb/IIIa (CD41/CD61), or GPIbIX (CD42b and CD42a), yielding no significant findings.

Hemolysis markers (Hb, LDH, and bilirubin) did not show evidence of ongoing hemolysis. However, as this association was not evaluated a priori, additional markers such as haptoglobin and reticulocyte count were not assessed.

A pharmacological evaluation concluded that the multifactorial pancytopenia was primarily due to a single cycle of gemcitabine administered on 30 June 2025. So, according to the pharmacologist’s initial hypothesis, the toxicity was correlated with gemcitabine toxicity. Currently, there are no evidence-based genetic signatures with which to assess gemcitabine-related toxicity.

A multigene panel evaluating the Excision Repair Cross-Complementing Group 1 (ERCC1) enzyme, the X-Ray Repair Cross-Complementing Group 1 (XRCC1) enzyme, and glutathione S-transferase P1 (GSTP1) may predict clinical outcome following platinum treatment. However, no mutations predictive of toxicity were detected.

In September 2025, the treatment response was evaluated according to EBV DNA level and the results of a CT-PET scan.

EBV DNA was undetectable.

**Figure 4:**
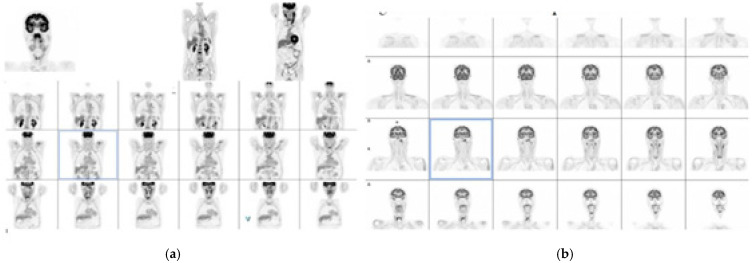
(**a**) CTPET scan at baseline in June 2025. (**b**) CTPET scan at baseline in September 2025.

The results of the CT-PET scan were compared to those of the baseline scan, and a complete response was detected. Figure 4 reports CTPET scan imagings.

In summary, this 58-year-old patient, with no significant comorbidities, exhibited an early and marked treatment response but also developed severe and prolonged toxicity, which precluded the planned treatment.

The response was evaluated according to EBV DNA reduction and CT-PET scan comparation (baseline (June) versus September). The PERCIST criteria were used to interpret the CT-PET scan (with a complete response defined as the disappearance of all metabolic activity). Although the target lesions and their sizes could be assessed according to the RECIST criteria, since the CT examination was conducted without contrast (basal CT associated with PET), the metabolic response was evaluated.

## 3. Discussion

The first classification of G6PD variants was published more than half a century ago, as reported in Table 3 [9].

However, the G6PD gene is highly polymorphic, and, to date, more than 230 variants are known. Table 4 presents the latest classification, which is designed to address the clinical need to classify heterozygous males as either class II or III [10].

Although typically asymptomatic, G6PD deficiency may present clinically as neonatal jaundice; rarely, it presents as acute hemolytic anemia triggered by fava bean ingestion, infections, or drugs. Very rarely, it may manifest as chronic non-spherocytic hemolytic anemia.

Indeed, the threshold of 10%—previously used to distinguish class II from class III variants—provided a strong argument for abandoning this separation and ultimately led to the revised WHO classification. Classes I and V have been retained due to their rarity. A recent consensus highlighted that the variability in enzyme activity values for the same variant may reflect biological factors, technical limitations, or methodological issues, such as white blood cells’ interference with red cell G6PD activity. The lack of reliable data for some variants underscores the need to generate more robust evidence on phenotype–genotype associations for each variant.

Glucose-6-phosphate dehydrogenase (G6PD) activity is elevated in cancer cells as a consequence of the Warburg effect. Reduced G6PD activity leads to premature cellular senescence and cell death due to oxidative stress, whereas aberrant activation promotes uncontrolled cell growth and altered differentiation [3,11].

Moreover, G6PD overexpression has been reported to contribute to chemotherapy and targeted therapy resistance in several solid tumors, including non-small-cell lung cancer, pancreatic cancer, colorectal cancer, breast cancer, and head and neck cancers [12,13,14].

Low G6PD expression correlates with high immune activity and may predict a favorable response to immune checkpoint blockade therapy. The mechanism linking G6PD to immune activity is still unclear. Because G6PD has a role in protecting cells from death, we speculate that its downregulation may induce oxidative-stress-mediated immunogenic cell death in cancer cells. This immunogenic cell death enhances antigen presentation, thereby activating antitumor immunity [15]. Cisplatin-resistant cells are more sensitive to G6PDH inhibition. High levels of G6PD correlate with poor prognosis, whereas cancer patients harboring G6PD mutation show longer survival and reduced metastases. Previous experiences have confirmed that the inhibition of G6PD may restore cancer cells’ sensitivity to chemotherapy [16,17].

G6PD blockade is a promising therapeutic strategy. G6PD inhibitors block the pentose phosphate pathway, thereby suppressing cancer cell proliferation and metastases. While G6PD inhibitors have demonstrated efficacy against several types of cancer, their combination with ICIs has not been reported.

Most reports affirm that there is no increased sensitivity or toxicity in platinum- treated patients with G6PD deficiency, nor is there increased hemolysis. Jiang et al. described a nasopharyngeal carcinoma patient treated with induction chemotherapy (docetaxel, cisplatin, cetuximab, and 5-Fluorouracil) followed by concurrent chemoradiotherapy with three doses of cisplatin (100 mg/m^2^), with no toxicities observed. The adverse events reported included grade 1 neutropenia, grade 1 diarrhea, and grade 2 acneiform skin reactions during induction chemotherapy and grade 1 neutropenia, grade 1 diarrhea, grade 2 oral mucositis, and grade 1 skin reactions during concomitant chemoradiotherapy [18].

These reports involve regimens without strong oxidative potential, limiting their comparability with platinum–gemcitabine.

La Verde et al. reported the safety of chemotherapy with docetaxel and cyclophosphamide in a G6PD-deficient early-breast-cancer patient. It must be stressed that neither docetaxel nor cyclophosphamide was predicted to elicit oxidative stress [19].

In a retrospective Sardinian study involving 40 Caucasian female patients with non-metastatic breast cancer and G6PD deficiency, the authors demonstrated the safety of anthracycline-based chemotherapy. Thirty percent of patients were treated with an FEC regimen (5-Fluoruracil (500 mg/m^2^ iv), Epirubicin (75–100 mg/m^2^ i.v.), and Cyclophosphamide (500–600 mg/m^2^ i.v.) every three weeks) in six cycles, and 70% of the women underwent chemotherapy, including anthracyclines and taxanes. A total of 55% were administered an EC regimen (Epirubicin (90 mg/m^2^ i.v.) and Cyclophosphamide (600 mg/m^2^ i.v.) every two weeks) in four cycles and Paclitaxel (80 mg/m^2^ i.v.) weekly for 12 cycles, while 15% were treated with a FEC regimen in three cycles and Docetaxel 100 mg/m^2^ i.v. every three weeks in three cycles. No safety concerns were reported, except for a slight increase in lactate dehydrogenase levels [20].

The use of rasburicase (a recombinant urate oxidase that catalyzes the oxidation of uric acid into allantoin) to prevent tumor lysis syndrome is contraindicated for GP6D deficient patients because of the drug’s potential to cause hemolytic anemia [21].

Data on anthracycles and platinum salts have been further analyzed, with several studies reporting no issues for either doxorubicin or epirubicin or platinum salts [22,23,24,25].

To avoid hemolytic complications in patients with an unknown G6PD status or when using drugs with unclear oxidative stress profiles, enhanced pharmacogenetic screening is essential.

In oncology, although few cases of rasburicase-induced hemolysis have been reported and only one case of an anthracycline-induced hemolytic event in G6PD-deficient patients has been described, awareness of chemotherapy outcomes in this population remains extremely limited in the literature [26].

Our patient developed severe grade 4 toxicity after 10 days from the first single cycle of chemotherapy. The Cisplatin Gemcitabine protocol is most closely associated with hematologic toxicity in patients with bone metastases; so, the observed toxicity may be multifactorial and cannot be attributed with certainty to G6PD deficiency alone. Other reports support the notion of platinum-based toxicity in other cancer patients (such as those with dyspnea, radiographic pulmonary infiltrates, and unexplained tachycardia while on both FOLFOX chemotherapy and febrile neutropenia underlying G6PD deficiency, or severe neutropenia in combination with 5 FU) [27,28].

Unfortunately, there are no prior reports describing cisplatin and gemcitabine treatment for a patient with G6PD deficiency and EBV-positive nasopharyngeal carcinoma. Cisplatin stops tumor growth by cross-linking the guanine bases within DNA double-helix strands, thereby directly damaging DNA.

Radiation therapy sensitivity may also be increased, potentially leading to enhanced responses to oxidative stress.

It must be stressed that due to the single-case nature of this report and the lack of G6PD genotyping re-testing and bone marrow evaluation, we cannot rule out the drug’s induced toxicities, such as gemcitabine-related myelotoxicity. We are aware that the absence of further molecular characterization and bone marrow histology are limitations.

## 4. Conclusions

In this report, we describe a case characterized by a rapid and sustained clinical response accompanied by severe toxicities.

This report should be interpreted as hypothesis-generating only, given its single-case nature. The absence of confirmatory testing for G6PD status represents an important limitation, as it precludes the drawing of definitive conclusions regarding the underlying biological susceptibility. Furthermore, no mechanistic validation was performed, and thus the proposed pathophysiological explanations remain speculative. Additional confounding factors, including the treatment regimen and the underlying disease burden, may also have influenced the observed outcome. Collectively, these limitations restrict the generalizability of the findings and underscore the need for further investigation in larger, systematically studied cohorts.

However, given that G6PD deficiency is relatively common, caution should be exercised when treating such patients with cytotoxic agents, particularly alkylating drugs.

Indeed, it is crucial to consider the possibility of an unusual drug sensitivity in patients who experience prolonged toxicities or exhibit an exceptional therapeutic response after a single chemotherapy cycle.

Alternative explanations pertain to, e.g., treatment efficacy, disease biology, and the additive radiotherapy effect (abscopal).

As stated above, the weaknesses of our report are the absence of bone marrow biopsy and reported genetic testing (which was not repeated to confirm asymptomatic G6PD variants), so we highlight the need for further studies rather than drawing definitive clinical implications. However, the strength of this case lies in the fact that, to our knowledge, this is the first report to hypothesize a personalized approach to cancer treatment for G6PD-deficient patients. Moreover, all imaging and laboratory assessments were performed at the same center, ensuring consistency of data.

Clinicians should carefully weigh the potential benefits and risks of cytotoxic therapies for patients with G6PD deficiency. Updated guidelines are needed to support oncologists and physicians in determining appropriate dosing and scheduling of chemotherapy in this population. Additionally, since a pharmacologist may not always be readily available in oncology wards, an on-demand consultation system should be considered.

## Figures and Tables

**Table 1:** Hematologic values observed during therapy.

	11/6/25	7/7/25 *	16/7/25 *	20/7/25 *	24/7/25 *	28/7/25 **	5/8/25	13/8/25	21/8/25 ***	28/8/25	1/9/25	5/9/25
LDH	237	191		241			278	248	217	236		
WBC	5.28 §	3.52 §	1.36 §	2.61 §		5.56 §	4.77 §	4.86 §	5.14 §	8.77 §	1.03 §	2.5 §
PLT	110 §	57 §	30 §	20 §		109 §	51 §	79 §	90 §	64 §	36 §	20 §
HB	12.8 g/L	11.8 g/L	11.3 g/L	9.3 g/L		9.5 g/L	9.5 g/L	9.3 g/L	10.8 g/L	10.7 g/L	11.1 g/L	
NEU	3.81 §	2.47 §	3.90 §	1.21 §		3.63 §	2.72 §	3.12 §	3.21 §	6.92 §	8.27 §	0.85 §
LIN	0.89 §	0.89 §	0.65 §	0.8 §		1.07 §	1.38 §	1.04 §	0.82 §	0.9 §	0.84 §	
EBVDNA	5470 Copy number	4557 Copy number			351 Copy number					<36 Copy number		

Abbreviations: LDH, lactate dehydrogenase; WBC, white blood cell; PLT, platelets; HB, hemoglobin; NEU, neutrophil; LIN, lymphocytes; EBVDNA, Epstein–Barr Virus DNA. § × 10^9^/L × 2nd cycle postponed starting on 2 July 2025. * 1st cycle. ** 2nd cycle. *** 1st weekly carboplatin.

**Table 2:** Common medications to be avoided or used with caution when treating G6PD-deficient patients.

Sulfonamides
Nitrofurans
Antimalarials—chloroquine, primaquine
Antipyretics
Dapsone
Probenacid
Aspirin
Chloramphenicol

**Table 3:** WHO classification of G6PD deficiency.

**Class I** **(Very Severe)**	Enzyme activity: <10% of normal
	Clinical features: Chronic non-spherocytic hemolytic anemia (CNSHA), with potential intermittent acute hemolysis
	Rare but clinically significant
**Class II** **(Severe)**	Enzyme activity: <10% of normal
	Clinical features: Intermittent acute hemolytic anemia, usually triggered by infections, certain drugs, or fava bean ingestion
	No chronic hemolysis in most cases
**Class III** **(Moderate)**	Enzyme activity: 10–60% of normal
	Clinical features: Intermittent hemolysis, usually only under oxidative stress (drugs, infections, and fava bean ingestion)
	Most common symptomatic group
**Class IV** **(Mild/Asymptomatic)**	Enzyme activity: 60–100% of normal
	Clinical features: Usually asymptomatic, no significant hemolysis
**Class V** **(Increased activity)**	Enzyme activity: >150% of normal
	Clinical features: No clinical consequence; usually detected incidentally

**Table 4:** Current WHO classification of GP6D variants.

G6PD Variant Class	Median G6PD Activity (% of Normal)	Associated Clinical Manifestations
A	<20% *	Chronic hemolytic anemia
B	<45%	Neonatal jaundice; acute hemolytic anemia triggered by certain drugs, fava beans, or infection
C	>60%	No hemolysis
U **	Any	Uncertain clinical significance

G6PD: glucose-6-phosphate dehydrogenase; WHO: World Health Organization. * A = <20% activity without acute hemolysis if symptoms are of Class B. ** Temporary classification if there is insufficient information regarding clinical manifestations.

## Data Availability

The original contributions presented in this study are included in the article. Further inquiries can be directed to the corresponding author.
